# Altitude-Dependent Differences in Non-Volatile Metabolites of Tea Leaves Revealed by Widely Targeted Metabolomics

**DOI:** 10.3390/biology15030224

**Published:** 2026-01-25

**Authors:** Jilai Cui, Yiwei Yang, Yu Che, Lumiao Yan, Qi Zhang, Qing Wei, Jie Li, Jie Zhou, Bin Wang

**Affiliations:** 1 Dabie Mountain Laboratory, College of Tea and Food Science, Xinyang Normal University, Xinyang 464000, China; 2 Henan Key Laboratory of Tea Plant Biology, College of Tea and Food Science, Xinyang Normal University, Xinyang 464000, China; 3 Henan International Joint Laboratory of Tea-Oil Tree Biology and High-Value Utilization, College of Tea and Food Science, Xinyang Normal University, Xinyang 464000, China; 4 Xinyang Academy of Agricultural Sciences, Xinyang 464000, China

**Keywords:** tea, metabolites, altitude, widely targeted metabolomics

## Abstract

Tea is produced from the fresh leaves of the tea plant (*Camellia sinensis*), and the quality of tea is directly dictated by its raw material. Although factors such as tea cultivar, fertilization, and cultivation practices are known to affect fresh leaf quality, the specific influence of altitude remains poorly understood. In this present study, ultra-performance liquid chromatography-tandem mass spectrometry (UPLC-MS/MS) was employed to investigate the non-volatile metabolites in fresh tea leaves grown at two different altitudes (350 m and 600 m). A total of 2323 metabolites were identified, with flavonoids and phenolic acids representing the dominant classes. Orthogonal partial least squares-discriminant analysis (OPLS-DA) further revealed 116 differential metabolites between groups. Kyoto Encyclopedia of Genes and Genomes (KEGG) pathway enrichment analysis indicated that several key pathways were differentially activated, including those related to the biosynthesis of kaempferol, luteolin, and flavones, as well as nucleotides and jasmonic acid metabolism. In addition, marked differences were observed in the accumulation patterns of lipids, phenolic acids, and flavonoids between leaves grown at the two altitudes. These findings provide valuable insights into the role of altitude in shaping the metabolic composition and flavor formation of tea.

## Simple Summary

Tea is a globally popular beverage, but little is known about how altitude affects its flavor and health benefits. This study investigated the chemical differences between fresh tea leaves grown at low altitudes (350 m) and high altitudes (600 m) using advanced analytical methods. We found significant metabolic differences between the two environments, identifying over 2300 compounds, including key flavor molecules like flavonoids and phenolic acids. High-altitude samples displayed a specialized metabolic signature marked by elevated levels of tannins and stress-induced flavonoids, diverging from low-altitude samples, which possessed higher lipid and total polyphenol contents. These results demonstrated that the traditional belief that “good tea is produced from high mountains” has a solid scientific basis. Our findings provide vital information for tea growers, helping them optimize cultivation methods at different elevations to produce tea with specific, enhanced flavor profiles and health properties.

## 1. Introduction

Tea is highly valued worldwide for its health-promoting properties and unique flavor profiles. Its diverse chemical composition, including polyphenols, caffeine, amino acids, and aroma compounds, not only provides health benefits such as antioxidant activity and mental stimulation but also contributes to its varied sensory characteristics [1,2,3,4]. These bioactive components originate from the fresh leaves and from the subsequent processing stages. Considerable research has therefore focused on elucidating the formation mechanisms of characteristic compounds during processing. For instance, in black tea production, polyphenols in fresh leaves undergo enzymatic oxidation and polymerization during fermentation, leading to the formation of theaflavins and thearubigins, which influence both the color and characteristic flavor of black tea while also contributing to its bioactivity [5,6]. Similarly, the shaking step in oolong tea processing physically disrupts leaf cells, initiating a series of enzymatic reactions that produce its signature floral and fruity aromas [7,8]. In contrast, despite these extensive investigations into processing-related transformations, the chemical composition of fresh tea leaves has received comparatively less attention. Given this research gap, it is essential to further investigate the intrinsic chemical composition of fresh tea leaves and the environmental factors that modulate it.

The metabolite composition of fresh tea leaves is strongly influenced by environmental conditions, with numerous studies demonstrating that factors such as light, temperature, fertilization, and altitude can significantly shape both leaf chemistry and overall tea quality. For example, research indicates that specific light qualities, such as blue and red light, can enhance the aroma of fresh tea leaves, while moderate shading can reduce polyphenol content, which is often linked to an improvement in tea quality [9,10]. In terms of temperature, studies have revealed that elevated temperatures can lead to a reduction in theanine levels, resulting in a deterioration of tea’s taste [11]. Fertilization also plays a crucial role: for instance, the combined application of chemical and organic fertilizers has been shown to promote the synthesis of catechins and flavonoids by regulating key genes in the flavonoid biosynthesis pathway, thereby enhancing overall tea quality [12,13]. Altitude represents another important factor influencing leaf metabolites. Although the traditional Chinese saying “good tea comes from high, misty mountains” has long suggested a positive role of altitude, the direct impact of elevation on the metabolite profile of fresh tea leaves remains insufficiently understood.

In the field of phytochemistry, traditional analytical methods such as high-performance liquid chromatography (HPLC), ultra-performance liquid chromatography-mass spectrometry (UPLC-MS), and ultra-performance liquid chromatography-quadrupole-time of flight-mass spectrometry (UPLC-Q-TOF-MS) have been extensively used for analyzing plant active ingredients. Rather than being viewed in isolation, the emergence of widely targeted metabolomics represents a significant advancement that leverages the complementary strengths and synergistic potential of these established techniques [14,15,16]. This integrated approach bridges the gap between untargeted discovery and targeted quantification. The application of multiple reaction monitoring (MRM) technology enhances the analytical platform by combining the high-resolution scanning capabilities of discovery-based metabolomics with the exceptional sensitivity, precision, and high throughput characteristic of targeted analysis [17,18]. Consequently, this synergistic methodology has been increasingly adopted to explore metabolic differences across diverse biological samples with superior accuracy and broad coverage. These include flavonoids and phenolic acids, which directly shape sensory characteristics such as taste [19], color [20], and nutritional profiles [21,22].

Despite the increasing use of advanced metabolomics, research utilizing widely targeted metabolomics based on UPLC-MS/MS to explore the differences in non-volatile metabolites of tea leaves at various altitudes remains limited. To bridge this gap, our study employed this advanced technique to systematically identify and compare the chemical compositions and differentially accumulated metabolites of tea leaves grown at two distinct altitudes. The findings from this research not only elucidate the metabolic pathways responsible for the formation of tea leaves under different altitude conditions but also provide a solid scientific foundation for optimizing tea cultivation practices and improving tea quality.

## 2. Materials and Methods

### 2.1. Chemicals and Regents

The liquid chromatography-mass spectrometry grade methanol and acetonitrile used in this experiment were purchased from Merck (Darmstadt, Germany), and formic acid was purchased from Aladdin (Shanghai, China).

### 2.2. Sample Collection and Extraction

Fresh tea shoots with one bud and two leaves were collected from Yuwu Mountain (Dongjiahe, Shihe district, Xinyang, China, 113°49′54″ E, 32°2′ N) on 15 April 2024. The plant material for all samples belonged to *Camellia sinensis* var. *sinensis* cv. Xinyang quntizhong is the primary landrace used for producing Xinyang Maojian green tea. Critically, both the low-altitude (LFT, approx. 350 m) and high-altitude (HFT, approx. 600 m) samples were sourced from adjacent tea gardens with identical soil type, fertilization regimes, and non-shading cultivation practices, ensuring that only the altitude and its associated climatic variables differed. The LFT and HFT collection sites share the same geographical coordinates (region) and were under the management of the same local tea farmers. For sampling, three independent biological replicates (*n* = 3) were collected for each altitude group (LFT and HFT). The 350 m and 600 m sites represent the typical commercial contrast between “plain tea” and “high-mountain tea” in Xinyang. Each biological replicate consisted of pooled fresh shoots collected randomly from ten healthy, mature tea plants within the respective altitude plot. Immediately after collection, all fresh tea shoots were flash-frozen in liquid nitrogen and subsequently vacuum freeze-dried.

The dried samples were ground into fine powder using a mixer mill (MM 400, Retsch GmbH, Haan, Germany; 30 Hz, 1.5 min). The metabolite extraction was performed following the methods [23] with minor modifications, 50 milligrams of powder were suspended in 1200 μL of a pre-chilled 70% methanol containing internal standards. The mixture was vortexed intermittently (30 s pulse at 30 min intervals, 6 cycles total) to maximize analyte recovery. Following centrifugation at 12,000 rpm for 3 min, the supernatant was filtered through a 0.22 μm membrane filter and transferred to LC-MS vials for UPLC-MS/MS analysis. In addition, 40 μL aliquots from each sample were pooled to generate quality control (QC) samples.

### 2.3. Metabolite Detection and Identification

The sample extracts were analyzed using a UPLC-MS/MS system (ExionLC AD system coupled with a QTrap 6500+ mass spectrometer, AB SCIEX, Framingham, MA, USA). The UPLC and mass spectrometry were conducted according to the previous methods with modifications [24]. Chromatographic separation was achieved using an Agilent SB-C18 column (Agilent Technologies, Santa Clara, CA, USA; 2.1 mm × 100 mm, 1.8 μm particle size) maintained at a constant temperature of 40 °C. The mobile phase consisted of (A) ultrapure water containing 0.1% (*v*/*v*) formic acid and (B) acetonitrile containing 0.1% (*v*/*v*) formic acid. A gradient elution program was applied as follows: 0.00–9.00 min, a linear increase from 5% to 95% B; 9.00–10.00 min, isocratic at 95% B; 10.00–11.10 min, a linear decrease to 5% B; followed by re-equilibration at 5% B until 14.00 min. The flow rate was set at 0.35 mL/min, and the injection volume was 2 μL.

Mass spectrometric detection was carried out using an electrospray ionization (ESI) source operated at 550 °C. The ion spray voltage was set at 5500 V in positive ion mode and −4500 V in negative ion mode. Source gas parameters were optimized as follows: nebulizer gas (GSI), auxiliary gas (GSII), and curtain gas (CUR) at 50, 60, and 25 psi, respectively. Collision-induced dissociation (CID) was activated at “high” energy level. Triple quadrupole (QQQ) scanning was conducted in MRM mode, using nitrogen as the collision gas at “medium” pressure. Declustering potential (DP) and collision energy (CE) were individually optimized for each MRM transition to maximize sensitivity. During data acquisition, specific MRM ion pairs were dynamically monitored within predefined retention time windows corresponding to metabolite elution.

### 2.4. Qualitative and Quantitative Principles of Metabolites

Metabolite identification was performed using the self-built Metware database (Metware Analyst Software; Version 1.0, Metware Biotechnology Co., Ltd., Wuhan, China) through a multi-dimensional matching process: (1) Precursor ion (Q1) and product ion (Q3) pairs in MRM mode; (2) Retention time (RT) matching; and (3) comparison of secondary mass spectrometry fragmentation patterns with the standard spectra in the database.

For metabolite quantification, the MRM mode was employed according to the previous method [25]. In this method, the first quadrupole isolates the precursor ions of target compounds, effectively excluding interfering ions from other substances. After collision-induced dissociation in the collision cell, the precursor ions fragment into numerous product ions. The third quadrupole then selects a specific characteristic fragment ion, which further minimizes non-target ion interference, thereby ensuring higher accuracy and reproducibility in quantification. After acquiring mass spectrometry data from all samples, all chromatographic peaks were integrated, and the peak areas of the same metabolite across different samples were integrated and corrected.

### 2.5. Statistical Analysis

Raw data were filtered, aligned and calculated using Analyst 1.6.3 software (AB SCIEX Pet. Ltd., Framingham, MA, USA). Unsupervised principal component analysis (PCA) was performed on unit variance-scaled data using R’s prcomp (version 4.3). Hierarchical cluster analysis (HCA) and Pearson correlation heatmaps were generated via the ComplexHeatmap package (version 2.14.0). Differential accumulated metabolites were identified by combining orthogonal partial least squares-discriminant analysis (OPLS-DA; Variable Importance in Projection, VIP > 1) with log2-fold change thresholds (|Log2FC| ≥ 1.0) and *p* < 0.05. The robustness of the OPLS-DA modes was validated through 200 permutation tests after log2 transformation and mean-centering [26]. Metabolites annotated using the Kyoto Encyclopedia of Genes and Genomes (KEGG) Compound database were mapped to their respective pathways. This was followed by a metabolite set enrichment analysis (MSEA), with statistical significance assessed using a hypergeometric test *p*-value.

## 3. Results and Discussion

### 3.1. Metabolites Identification in LFT and HFT

**Figure 1:**
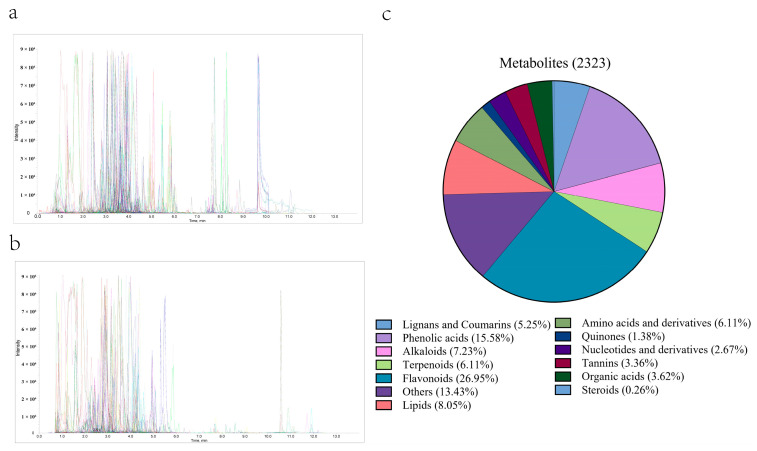
Metabolite identification in tea samples. Representative total ion chromatogram by the widely targeted metabolomics approach in positive mode (**a**) and negative mode (**b**). Types and proportions of the metabolites identified (**c**).

To investigate the metabolic difference between fresh tea leaves grown at low-altitude (LFT) and high-altitude (HFT), widely targeted metabolomics were employed. Representative MRM chromatograms of QC samples obtained by widely targeted metabolomics are shown in Figure 1 (Figure 1a, positive mode; Figure 1b, negative mode). As can be seen from the figures, all target compounds exhibited symmetrical chromatographic peaks, and satisfactory chromatographic separation of individual target compounds was well achieved. Metabolites were quantitatively analyzed based on secondary spectral data acquired in MRM mode, leading to the identification of 2323 metabolites. These metabolites were classified into 13 categories, including 626 flavonoids, 362 phenolic acids, 187 lipids, 168 alkaloids, 142 amino acids and derivatives, 142 terpenoids, 122 lignans and coumarins, 78 organic acids, 78 tannins, 62 nucleotides and derivatives, 32 quinones, 6 steroids, and 312 others. Among these, flavonoids (26.95%), phenolic acids (15.58%), lipids (8.05%), alkaloids (7.23%), amino acids and their derivatives (6.11%) and terpenoids (6.11%) represented the six dominant metabolite classes (Figure 1c), providing a comprehensive overview of the metabolic landscape in tea leaves from different altitudes.

### 3.2. Multivariate Analysis of Identified Metabolites in LFT and HFT

**Figure 2:**
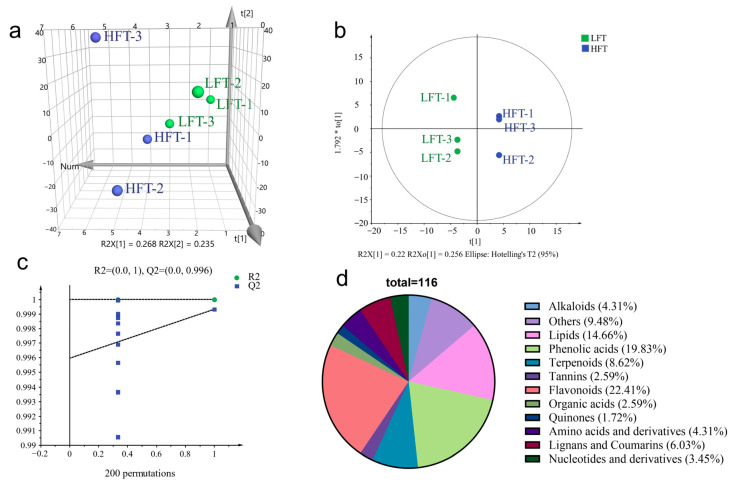
Analysis of differential metabolites (**a**) Scores plots of PCA, (**b**) Scores plots of OPLS-DA (R^2^X = 0.396, R^2^Y = 0.997, Q^2^ = 0.445), (**c**) cross-validation plot by 200 permutation tests (R^2^ = 1, Q^2^ = 0.996), (**d**) Proportion of categories of differential compounds.

To understand the overall metabolic differences between LFT and HFT, a principal component analysis (PCA) was first conducted. The PCA demonstrated that although the between-group variation was relatively small, LFT and HFT samples could still be clearly separated in the 3D-PCA score plot (Figure 2a). The first two principal components (PC1, 26.8%; PC2, 23.5%) together accounted for 50.3% of the total variance, demonstrating the significant contribution of these components to the observed metabolic variation. To further enhance group discrimination and obtain a more robust evaluation of metabolic variation, an OPLS-DA model was subsequently applied.

The OPLS-DA model revealed a clear separation between the two groups (Figure 2b). The prediction parameters of the OPLS-DA model were R^2^X, R^2^Y, and Q^2^, with values closer to 1 indicating higher model stability and reliability. Meanwhile, both R^2^Y and Q^2^ exceeded 0.9, suggesting excellent model performance. To confirm the robustness of this model, a 200-time permutation test was conducted, further validating the realism and excluding the possibility of overfitting (R^2^ = 1, Q^2^ = 0.996) (Figure 2c).

Based on this validated model, a total of 116 differential metabolites were identified between HFT and LFT, using the thresholds of VIP > 1, *p*-value < 0.01, and |Log2FC| ≥ 1.0 (Table 1). These metabolites, representing diverse chemical classes, highlight the compositional shifts in tea leaves associated with altitude. They were categorized into 12 groups, with flavonoids (22.41%), phenolic acids (19.83%), and lipids (14.66%) being the predominant categories (Figure 2d).

### 3.3. Analysis of Differential Metabolites

**Figure 3:**
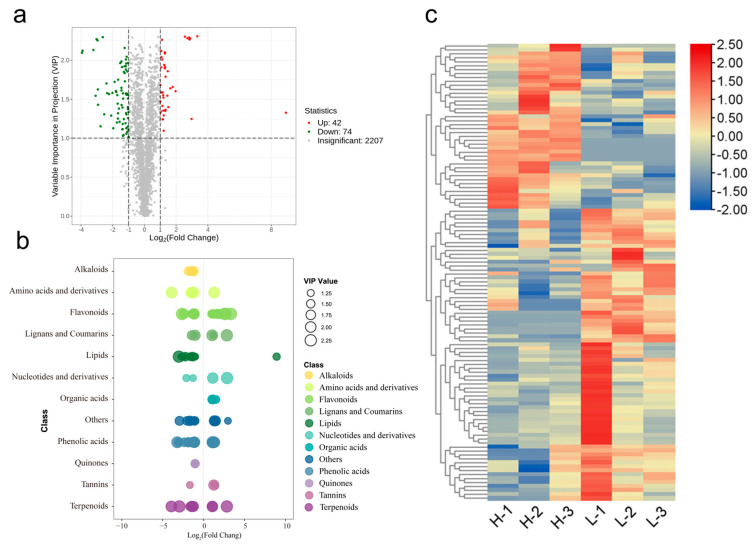
Analysis of differential metabolites (**a**) Volcanic diagram of differential metabolites. Red color indicates up-regulated compounds, and green color indicates down-regulated compounds. (**b**) Categories of differential metabolites—content scatter plots, the larger circle means the greater VIPs obtained from the OPLS-DA model. (**c**) Cluster heat map of differential metabolites. H-1, H-2, and H-3 were samples from HFT group and L-1, L-2, and L-3 were samples from LFT group. Red color means higher content.

After validating the robustness of the OPLS-DA model, we next focused on characterizing the specific metabolites that contributed to the observed differences between LFT and HFT. The differential metabolites obtained from the screening were analyzed in terms of their upregulation and downregulation patterns. As shown in Figure 3a, a total of 116 differential metabolites were identified between LFT and HFT, of which 42 were upregulated and 74 were downregulated (Figure 3a, Table 2). These metabolites were primarily distributed across polyphenols, amino acids and derivatives, lipids, terpenoids, alkaloids, nucleotides and derivatives, and organic acids (Figure 3b,c). To visualize the distribution of these compounds, a heatmap was generated (Figure 3c), which clearly shows that all differential lipids were more abundant in LFT, suggesting a notable influence of altitude on lipid metabolism in tea leaves.

Our findings demonstrate that even a relatively narrow altitudinal gradient (approx 250 m) can serve as a potent environmental filter. This is consistent with the ‘high mountain produces good tea’ theory in traditional Chinese tea science, where subtle changes in temperature and light quality at these specific elevations are sufficient to trigger the accumulation of stress-related metabolites, thereby shaping the unique flavor profile of high-altitude tea. To further clarify the biological significance of these metabolic shifts, we systematically analyzed representative metabolite classes.

#### 3.3.1. Polyphenols

Polyphenols are a major group of secondary metabolites in tea, and they mainly composed of flavonoids, tannins, phenolic acids, and anthocyanins. In the present study, a total of 52 polyphenols were identified as differential metabolites. The overall abundance of polyphenols in low-altitude tea (LFT) was 1.6-fold higher than that in high-altitude tea (HFT). Within the flavonoid subgroup, 15 compounds were upregulated while 11 were downregulated. Among the three identified tannins, ellagic acid and theaflagallin were significantly enriched in HFT, exhibiting 2.5-fold and 2.4-fold higher concentrations compared to LFT, respectively. Regarding phenolic acids, 7 out of 23 were upregulated, with 1-O-Caffeoyl-6-O-galloyl-β-D-glucose showing the most pronounced increase (2.7-fold in HFT compared to LFT). Conversely, 16 phenolic acids were downregulated, and isotachioside displayed the most significant reduction, with its concentration in LFT being 9.3-fold higher than that in HFT.

**Figure 4:**
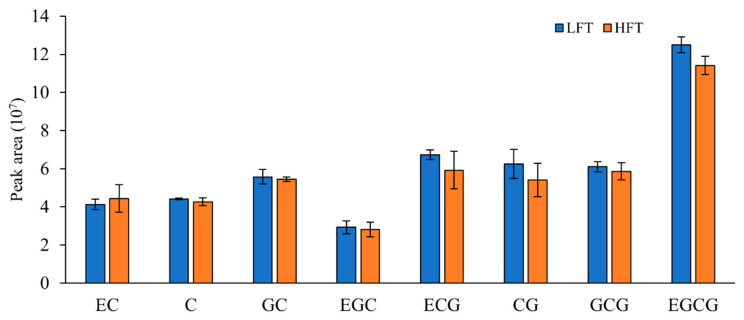
Relative content of main catechins in LFT and HFT. EC: Epicatechin, C: Catechin, GC: Gallocatechin, EGC: Epigallocatechin, ECG: Epicatechin gallate, CG: Catechin gallate, GCG: Gallocatechin gallate, EGCG: Epigallocatechin-3-O-gallate.

We acknowledge the critical role of catechins, such as Epigallocatechin gallate (EGCG), Epicatechin (EC), Epigallocatechin (EGC), and Gallocatechin (GC), as the principal determinants of astringency, bitterness, and antioxidant capacity in green tea [27]. Our quantitative analysis of these core catechin markers revealed that their concentrations were statistically similar between the LFT and HFT samples (Figure 4). For example, the most abundant catechins, EGCG and GCG, showed minimal differences in peak areas, and the total catechin content was statistically comparable across both altitudes. This stability suggests that the baseline quality profile, as defined by these major compounds, was successfully maintained across both altitudes due to controlled cultivation practices and the single cultivar used. Therefore, the overall metabolic differences observed primarily reflect specialized responses to altitude-induced stress, rather than shifts in the core quality profile.

Previous studies have demonstrated that phenolic acids are positively correlated with the bitterness of green tea infusions, and variations in their content directly influence the sensory intensity of bitterness, thereby reshaping the overall taste profile of tea [28]. The higher levels of phenolic acids in LFT may therefore lead to a more pronounced bitter and astringent taste, although their interactions with other flavor-active compounds could also contribute to a more balanced and harmonious flavor profile. In addition to their impact on taste, polyphenols—particularly flavonoids—are recognized as key contributors to the health-promoting properties of tea [29]. Flavonoids possess potent antioxidant activities, scavenging free radicals and mitigating oxidative stress-induced cellular damage, while also exerting protective effects on the cardiovascular system through mechanisms such as lipid metabolism regulation and anti-inflammatory pathways [30,31]. Moreover, certain flavonoid subtypes, including kaempferol and luteolin, have been implicated in modulating immune function and enhancing resistance against pathogens [32]. Interestingly, despite the lower overall polyphenol content in HFT compared to LFT, the number of upregulated flavonoid species (15) exceeded that of downregulated ones (11). Additionally, tannins with strong antioxidant activity were more abundant in HFT [33].

The significant accumulation of specific flavonoids (e.g., quercetin-type flavonols) in HFT samples suggests a robust secondary metabolic response to high-altitude stressors. At elevated solar irradiance typical of 600 m, these compounds act as internal UV-filters by absorbing harmful wavelengths and also serve as potent reactive oxygen species (ROS) scavengers, mitigating oxidative damage induced by increased UV exposure and high light intensity [34,35].

#### 3.3.2. Amino Acids and Their Derivatives

Amino acids are not only crucial contributors to the fresh and brisk taste of tea infusions but also play an essential role in shaping their overall aroma quality [36]. The present research identified 5 differential amino acids and derivatives between the two groups. Among them, homoproline showed a markedly higher accumulation in high-altitude tea leaves, reaching 2.4 times the level found in low-altitude samples. In contrast, the remaining four amino acids and their derivatives were more abundant in low-altitude tea leaves. As a unique non-protein amino acid found exclusively in tea plants, L-theanine is the primary contributor to the fresh and brisk taste of tea infusions [37]. The ratio of amino acids to EGCG plays a critical role in defining the sensory profile of tea. Analysis of this ratio revealed that tea samples from high-altitude regions exhibit a more balanced proportion, which helps mitigate the astringency associated with EGCG and promotes greater harmony in the overall flavor of the infusion.

Beyond their direct contributions to taste and aroma, amino acids are essential for tea plant growth and stress adaptation. They serve as precursors for functional molecules such as enzymes that drive metabolic pathways, and structural proteins that reinforce cell walls. From a sensory perspective, specific amino acids—particularly theanine—are known to mitigate the bitterness imparted by polyphenols, thereby enhancing the mellow and refreshing character of tea infusions [38,39]. Moreover, under environmental stress, amino acids function as osmolytes, helping to maintain cell turgor and stabilize biological membranes, which in turn supports plant survival in harsh conditions and influences the accumulation of other metabolites [40].

#### 3.3.3. Lipids

Among the 17 differential lipid metabolites identified, only one exhibited an upward trend at high altitudes. The remaining 16 lipids showed significantly higher levels in LFT. Notably, the content of 1-linoleoyl-2-lysophosphatidic acid monobutylamine ester in low-altitude samples was 8.6 times higher than that in HFT, highlighting the substantial influence of altitude on lipid accumulation.

As essential components of tea cell membranes, lipids play a critical role in maintaining membrane structure and fluidity. The observed regional differences in lipid content are likely linked to temperature variations associated with altitude [41]. Warmer conditions at low altitudes may promote lipid biosynthesis to sustain membrane fluidity, while cooler temperatures at high altitudes could suppress lipid accumulation by inhibiting lipid synthase activity [42,43]. Beyond structural functions, lipids also serve as important energy storage molecules. The more favorable thermal conditions at low altitudes provide abundant energy sources for lipid synthesis, thereby explaining the significantly higher total lipid content in low-altitude tea leaves. Moreover, variations in lipid metabolism may influence the uptake and transport of nutrients in tea plants. In particular, the elevated lipid content in LFT may enhance the utilization efficiency of fat-soluble nutrients in the soil, further supporting plant growth and metabolic activity [44,45].

#### 3.3.4. Terpenoids

Among the 10 differential terpenoid metabolites identified, 3 were upregulated while 7 were downregulated in LFT.

The biosynthesis of terpenoids is closely associated with the environmental adaptation strategies of plants. In high-altitude regions, strong ultraviolet (UV) radiation may stimulate the expression of key enzymes in terpenoid biosynthetic pathways [46,47]. Due to their molecular structures rich in conjugated double bonds, terpenoids can effectively absorb UV radiation, thereby protecting tea leaf cells from oxidative damage. Beyond their protective role, terpenoids are also crucial contributors to tea aroma. The upregulated terpenoids detected in high-altitude tea leaves may confer distinctive floral and fruity notes, which are often perceived as desirable sensory attributes [48].

#### 3.3.5. Lignans and Coumarins

Among the seven differential metabolites classified as lignans and coumarins, four were upregulated in HFT, while three were upregulated in LFT. Notably, isohydroxymatairesinol exhibited the most pronounced difference, with its concentration in HFT being 7.2 times higher than that in LFT.

Lignans are important structural components of plant cell walls, and their elevated levels can enhance the mechanical strength of tea leaf cell walls, which is particularly beneficial for tea trees growing at high altitudes to withstand mechanical stresses such as strong winds [49]. In contrast, coumarins are bioactive secondary metabolites with diverse physiological activities, including antioxidant and antibacterial properties, which may contribute to both plant defense and the functional value of tea [50].

#### 3.3.6. Alkaloids

All five alkaloids identified in this study exhibited a downward trend in HFT. Among them, the most pronounced difference was observed for (E)-S-(3-(4-hydroxy-3,5-dimethoxyphenyl) allyl) cysteine, whose concentration in LFT was 1.7 times higher than that in HFT.

Previous studies have reported that tea plants cultivated at lower altitudes generally accumulate higher levels of alkaloids due to more favorable growth conditions, resulting in infusions with stronger bitterness and greater irritancy compared to those from higher altitudes [51]. Conversely, in high-altitude regions, environmental stressors such as low temperatures and intense ultraviolet radiation suppress caffeine biosynthesis, leading to reduced alkaloid accumulation. As a result, tea from these regions tends to exhibit a softer taste profile with diminished bitterness. Collectively, these findings highlight the critical role of altitude in modulating alkaloid metabolism, particularly caffeine, thereby shaping the sensory characteristics of tea [52].

#### 3.3.7. Nucleotides and Derivatives

Among the four differential nucleotides and their derivatives detected, two were upregulated in HFT, while the other two showed higher levels in LFT. Specifically, flavin mononucleotide (FMN) and guanosine 3′,5′-cyclic monophosphate were elevated in HFT, with concentrations 1.3- and 1.6-fold higher, respectively, than those in LFT.

Nucleotides and their derivatives are fundamental to tea plant physiology, serving as central players in energy metabolism, nutrient transport, and the biosynthesis of flavor and functional compounds. They also act as essential building blocks of DNA and RNA, thereby regulating enzyme synthesis and influencing trait expression. Notably, FMN upregulation at high altitudes may enhance redox reactions and promote more stable photosynthetic activity under stressful environmental conditions [53].

#### 3.3.8. Organic Acids

Three organic acids were upregulated in high-altitude tea leaves, namely 2-trans-abscisic acid D-glucosyl ester, 3-hydroxybutyric acid, and jasmonic acid. Organic acids not only participate in respiratory metabolism, but also play critical roles in regulating intracellular pH and maintaining ion homeostasis in tea plants [54]. The modulation of jasmonic acid levels observed in our study points to the activation of hormone-mediated defense signaling. Jasmonic acid (JA) serves as a critical signaling hub that coordinates the metabolic shift towards the synthesis of protective secondary metabolites under environmental stress, thereby enhancing the plant’s adaptive plasticity to complex microclimates. Recent evidence indicates that JA and related jasmonates trigger the biosynthesis of flavonoids, phenylpropanoids, terpenoids, and other antioxidant compounds under abiotic stress conditions, coupling hormonal signaling with stress-responsive secondary metabolism [55,56].

In summary, altitude exerts a profound influence on the accumulation patterns of diverse metabolite classes in tea plants. Amino acids, flavonoids, lignans/coumarins, nucleotides, and organic acids tend to accumulate at higher altitudes, potentially enhancing stress resistance and conferring unique sensory qualities. By contrast, lipids and alkaloids are more abundant in low-altitude areas, likely due to favorable thermal conditions that promote membrane lipid synthesis and alkaloid accumulation, thereby intensifying bitterness. Terpenoids exhibit a mixed trend, with certain upregulated compounds in high-altitude teas contributing to distinctive floral and fruity aromas. Collectively, these results highlight that the environmental conditions shaped by altitude differentially regulate metabolic pathways, thereby driving both the adaptive physiology and the flavor formation of tea leaves.

### 3.4. KEGG Annotation and Enrichment Analysis of Differential Metabolism

To further elucidate the biological significance of the differentially accumulated metabolites (DAMs) between LFT and HFT, we employed a synergistic analytical framework integrating the KEGG pathway database and MetMap topology analysis. While KEGG provided the foundational mapping of metabolites to known biochemical reactions [57], MetMap was utilized to perform a more robust pathway topology analysis. Unlike traditional enrichment analysis that relies solely on the count of differential metabolites, MetMap evaluates the structural importance of each metabolite within the global metabolic network by calculating its Impact Value. This value is derived from the ‘centrality’ (including degree and betweenness centrality) of the DAMs, identifying ‘hub’ pathways that are more likely to represent critical physiological shifts in response to altitudinal gradients. By combining the enrichment significance (*p*-value) with the topology impact, we were able to pinpoint the most influential metabolic checkpoints modulated by altitude.

**Figure 5:**
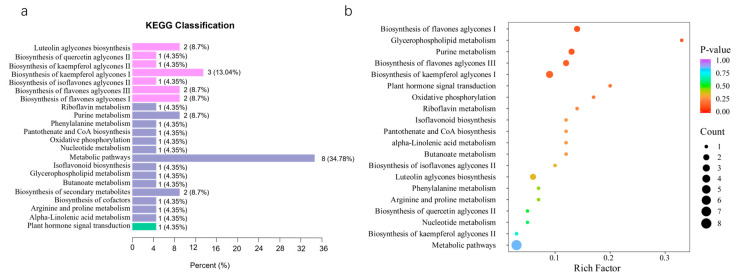
KEGG pathway annotation and enrichment analysis results of differential metabolites (**a**) KEGG pathway category distribution of differential metabolites, (**b**) KEGG pathway enrichment bubble plot for differential metabolites.

In our investigation, KEGG and MetMap functional annotations identified 22 metabolic pathways, involving 23 annotated differential metabolites, although the number of metabolites varied across pathways (Table 3). The annotation results were classified into three categories: MetMap, Metabolism, and Environmental Information Processing. MetMap represents extended pathway information, while Metabolism constituted the largest category, encompassing diverse substance metabolism pathways and accounting for the highest proportion of metabolite annotations (34.78%). In contrast, Environmental Information Processing, which reflects pathways associated with plant responses to external environmental cues, represented only a minor fraction (Figure 5a).

Enrichment analysis further revealed that several pathways were significantly enriched, notably the biosynthesis of kaempferol aglycones I–III, glycerophospholipid metabolism, and purine metabolism. Among these, glycerophospholipid metabolism exhibited the highest enrichment degree (Figure 5b), underscoring its central role in mediating altitude-related metabolic adjustments in tea leaves.

The prominence of glycerophospholipid metabolism is particularly noteworthy. As primary components of cellular membranes, glycerophospholipids undergo dynamic remodeling to maintain membrane fluidity and functional integrity under the fluctuating temperatures and cooler conditions characteristic of high-altitude environments, representing a key acclimation strategy in plants [58]. Similarly, the enrichment of the kaempferol aglycone biosynthesis pathway reflects a targeted photoprotective response, as kaempferol and other flavonoids function as efficient UV-absorbing compounds and reactive oxygen species scavengers that mitigate oxidative pressure induced by elevated UV-B radiation at higher elevations [59,60].

Overall, these results demonstrated that altitude strongly influences not only the accumulation of specific metabolites but also their distribution across diverse metabolic pathways, thereby shaping both the physiological adaptation of tea plants and the formation of key quality-related traits.

## 4. Conclusions

Using UPLC–MS/MS-based, widely targeted metabolomics, this study systematically profiled the metabolites of tea leaves grown at different altitudes. A total of 2323 metabolites were identified and classified into 13 categories, with flavonoids, phenolic acids, and lipids being the dominant groups. Multivariate statistical analysis (OPLS-DA) revealed distinct metabolic differences between high- and low-altitude tea, leading to the identification of 116 differential metabolites (42 upregulated and 74 downregulated in LFT). Pathway annotation and enrichment analysis indicated that these metabolites were mainly associated with flavonoid biosynthesis, glycerophospholipid metabolism, and purine metabolism, suggesting that altitude strongly affects both the accumulation of key metabolites and their related metabolic networks. In summary, this work provides a comprehensive overview of altitude-driven metabolic variations in tea, offering new insights into the biochemical mechanisms underlying tea quality formation. These findings not only contribute to a deeper understanding of environmental influences on tea chemistry but also provide a scientific basis for improving tea cultivation and processing practices.

## Figures and Tables

**Table 1:** Differential accumulated metabolites between HFT and LFT.

NO	Compounds	Class	CAS	VIP	*p*-Value	FC	Log_2_FC
1	(14-Ethyl-4,6,19-trimethoxy-9,11-dioxa-14-azaheptacyclo [10.7.2.12,5.01,13.03,8.08,12.016,20]docosan-21-yl)acetate	Alkaloids	N.F.	1.51	0.31	0.39	−1.34
2	(1s)-1-[(4-hydroxyphenyl)methyl]-7-methoxy-1,2,3,4-tetrahydroisoquinolin-8-ol	Alkaloids	N.F.	1.60	0.16	0.29	−1.81
3	(3R,4S,6R)-p-Menth-1-ene-3,6-diol-3-O-β-D-glucoside	Others	N.F.	1.92	0.02	2.48	1.31
4	(E)-S-(3-(4-Hydroxy-3,5-dimethoxyphenyl)allyl)cysteine	Alkaloids	N.F.	1.61	0.24	0.37	−1.45
5	1-(((2-(dimethylamino)ethoxy)(hydroxy)phosphoryl)oxy)-3-hydroxypropan-2-yl (9Z,12Z,15E)-octadeca-9,12,15-trienoate	Lipids	N.F.	1.38	0.34	0.33	−1.61
6	1-(2,3-dihydroxypropoxy)-3-(((2-(dimethylamino)ethoxy)(hydroxy)phosphoryl)oxy)propan-2-yl (8E,11Z,14Z)-octadeca-8,11,14-trienoate	Lipids	N.F.	1.20	0.36	0.30	−1.74
7	1-Galloyl-6-O-Benzoyl Glucose	Phenolic acids	N.F.	1.72	0.18	0.27	−1.88
8	1-Hydroxylup-20(29)-en-3-one (Glochidonol)	Terpenoids	23963-54-4	1.98	0.09	0.37	−1.45
9	1-Linoleoyl-2-Lysophosphatidic Acid Monobutylamine Ester	Lipids	N.F.	1.32	0.37	0.32	−1.67
10	1-Methoxybenzoyl-6-O-Galloyl-D-Glucose	Tannins	N.F.	1.25	0.37	0.31	−1.68
11	1-O-Caffeoyl-(6-O-glucosyl)-β-D-glucose	Phenolic acids	N.F.	1.59	0.30	0.29	−1.78
12	1-O-Caffeoyl-6-O-galloyl-β-D-glucose	Phenolic acids	N.F.	1.55	0.21	2.73	1.45
13	1-O-Caffeoylglycerol	Phenolic acids	N.F.	1.57	0.19	0.44	−1.17
14	1-O-Galloyl-3-O-Feruloyl-β-D-glucose	Phenolic acids	N.F.	1.34	0.27	2.06	1.04
15	1-O-Galloyl-4-O-p-Coumaroyl-β-D-glucose	Phenolic acids	N.F.	1.89	0.13	2.45	1.29
16	1-O-Galloyl-6-O-p-Coumaroyl-β-D-glucose	Phenolic acids	N.F.	1.68	0.17	2.00	1.00
17	1-O-p-Coumaroylsedoheptulose	Phenolic acids	N.F.	2.04	0.04	0.46	−1.12
18	1-Trans-5-trans-germacrone	Terpenoids	N.F.	2.04	0.08	2.11	1.08
19	2,3,5-trimethoxy-1-{[(2s,3r,4s,5s,6r)-3,4,5-trihydroxy-6-(hydroxymethyl)oxan-2-yl]oxy}xanthen-9-one	Flavonoids	N.F.	1.36	0.23	2.51	1.33
20	2,5,8-trimethylnona-1,4,7-triene-1,9-diol 1-O-Glyceryllinolenate	Others	N.F.	1.62	0.19	0.13	−2.94
21	2-(2,3-dihydroxypropoxy)-3-(((2-(dimethylamino)ethoxy)(hydroxy)phosphoryl)oxy)propyl (8E,11Z,14Z)-octadeca-8,11,14-trienoate	Lipids	N.F.	1.57	0.31	0.21	−2.23
22	2-Methyl-3-hydroxyindan-1-one	Others	N.F.	1.39	0.34	0.38	−1.38
23	2-trans-abscisic acid D-glucosyl ester	Organic acids	N.F.	1.59	0.24	2.22	1.15
24	2R-Hydroxyoctadecanoic Acid	Lipids	26633-48-7	2.27	0.05	0.12	−3.02
25	3′-Methoxydaidzin	Flavonoids	200127-80-6	1.41	0.19	2.19	1.13
26	3′-O-Methyl-6-hydroxygallocatechin 3-O-(2-Amino-5-hydroxypentanoate)	Flavonoids	N.F.	1.60	0.31	3.90	1.96
27	3,4-di-hydroxyphenethyol alcohol 4-O-β-D-(6′-O-galloyol)-glucopyranoside	Phenolic acids	N.F.	1.96	0.02	0.45	−1.15
28	3,6-dihydroxy-4,5-dimethoxy-1-{[(3r,4s,5s,6r)-3,4,5-trihydroxy-6-(hydroxymethyl)oxan-2-yl]oxy}xanthen-9-one	Flavonoids	N.F.	2.30	0.03	0.16	−2.63
29	3,8-Dihydroxy-1-methoxy-9,10-anthraquinone glucoside	Quinones	N.F.	1.40	0.38	0.48	−1.05
30	3-(((2-(dimethylamino)ethoxy)(hydroxy)phosphoryl)oxy)-2-hydroxypropyl (9Z,12Z,15E)-octadeca-9,12,15-trienoate	Lipids	N.F.	1.31	0.39	0.17	−2.54
31	3-(((2-(dimethylamino)ethoxy)(hydroxy)phosphoryl)oxy)-2-hydroxypropyl oleate	Lipids	N.F.	1.11	0.41	0.39	−1.38
32	3-Hydroxy-1-(4-Hydroxy-3-Methoxyphenyl)Propan-1-One	Others	2196-18-1	1.75	0.08	0.32	−1.66
33	3-Hydroxy-5-Methoxyphenyl-6-O-Galloyl-Beta-D-Galactopyranoside	Others	N.F.	1.57	0.19	0.47	−1.10
34	3-Hydroxy-5-Methylphenol-1-O-Glucoside	Others	N.F.	1.04	0.41	0.40	−1.32
35	3-Hydroxybenzoic acid	Phenolic acids	N.F.	1.86	0.14	0.47	−1.08
36	3-Hydroxybutyric acid	Organic acids	300-85-6	1.59	0.21	2.08	1.05
37	3-Hydroxycinnamic Acid	Phenolic acids	14755-02-3	1.59	0.16	2.08	1.06
38	3-Hydroxyphloretin-4′-O-[4″,6″-O-(S)-HHDP]-β-D-glucoside	Flavonoids	N.F.	1.66	0.08	3.44	1.78
39	4-Hydroxyacetophenone	Others	99-93-4	1.37	0.40	0.49	−1.03
40	4-Hydroxybenzoyl-1-O-(6″-O-galloyl)glucoside	Phenolic acids	N.F.	1.55	0.26	0.31	−1.71
41	4-O-Galloylarbutin	Phenolic acids	N.F.	1.56	0.09	0.49	−1.02
42	5,6-Dihydroxylucidin-3β-primeveroside	Quinones	N.F.	1.51	0.29	0.49	−1.04
43	6-Hydroxyluteolin	Flavonoids	18003-33-3	2.28	0.04	6.56	2.71
44	8-Epiloganic acid	Terpenoids	82509-41-9	2.25	0.05	0.13	−2.96
45	Acacetin-7-O-galactoside	Flavonoids	N.F.	1.55	0.14	2.26	1.18
46	Carthamidin	Flavonoids	479-54-9	1.33	0.45	2.02	1.01
47	Catechin glucosyl glucoside	Flavonoids	N.F.	2.30	0.01	5.88	2.56
48	Cis-L-3-hydroxyproline	Amino acids and derivatives	567-35-1	2.12	0.26	0.07	−3.91
49	D-Allo-Isoleucine	Amino acids and derivatives	1509-35-9	1.08	0.43	0.47	−1.09
50	D-Panthenol	Others	81-13-0	1.25	0.42	7.86	2.97
51	Dalbergin glucoside	Lignans and Coumarins	N.F.	1.24	0.38	2.21	1.15
52	Dihydroxybenzoyl xyloside	Phenolic acids	N.F.	1.18	0.38	0.39	−1.37
53	Ellagic acid	Tannins	476-66-4	1.35	0.36	2.50	1.32
54	Ent-16α,17-Dihydroxykauran-2-one	Terpenoids	N.F.	2.27	0.06	7.18	2.84
55	Epigoitrin	Others	1072-93-1	1.86	0.02	2.77	1.47
56	Eupatilin-7-O-glucoside	Flavonoids	N.F.	1.35	0.23	2.29	1.20
57	Flavin Single Nucleotide	Nucleotides and derivatives	6184-17-4	2.01	0.07	2.18	1.13
58	Formononetin-7-O-glucoside	Flavonoids	486-62-4	1.64	0.10	3.10	1.63
59	Furanodienone	Terpenoids	24268-41-5	1.92	0.07	0.42	−1.26
60	Gallic acid-1-O-xyloside	Phenolic acids	N.F.	1.32	0.18	0.45	−1.16
61	Gallic acid-3-O-(6′-O-galloyl)glucoside	Phenolic acids	87087-61-4	1.13	0.39	0.26	−1.97
62	Germacrone	Terpenoids	6902-91-6	1.91	0.12	2.10	1.07
63	Guanine	Nucleotides and derivatives	73-40-5	1.29	0.39	0.23	−2.10
64	Guanosine 3′,5′-cyclic monophosphate	Nucleotides and derivatives	7665-99-8	2.28	0.03	7.32	2.87
65	Homoproline	Amino acids and derivatives	56879-46-0	2.09	0.04	2.44	1.29
66	Hydroxymethyl coumarin glucoside	Lignans and Coumarins	N.F.	1.11	0.32	0.50	−1.01
67	Hydroxytyrosol	Others	10597-60-1	1.79	0.03	2.52	1.33
68	Icariside E5	Lignans and Coumarins	126176-79-2	1.65	0.18	0.37	−1.45
69	Isoguanine	Nucleotides and derivatives	3373-53-3	1.24	0.37	0.41	−1.30
70	Isohydroxymatairesinol	Lignans and Coumarins	N.F.	2.29	0.01	7.17	2.84
71	Isotachioside	Phenolic acids	31427-08-4	2.13	0.19	0.11	−3.22
72	Jasmonic acid	Organic acids	77026-92-7	1.40	0.25	2.84	1.51
73	Kaempferol 3-O-beta-D-(p-coumaroyl)-sambubioside	Flavonoids	N.F.	1.70	0.30	0.19	−2.40
74	Kaempferol-3-O-(2″-galloyl)galactoside	Flavonoids	N.F.	1.88	0.08	0.45	−1.17
75	Kaempferol-3-O-(6″-galloyl)galactoside	Flavonoids	N.F.	1.92	0.07	0.44	−1.18
76	Kaempferol-3-O-(6″-galloyl)glucoside	Flavonoids	56317-05-6	2.01	0.03	0.44	−1.17
77	Kaempferol-3-O-glucuronide-7-O-glucoside	Flavonoids	N.F.	1.42	0.30	0.37	−1.44
78	KakkaSaponin II	Terpenoids	N.F.	1.91	0.07	0.37	−1.42
79	L-Homonorleucine	Amino acids and derivatives	44902-02-5	2.16	0.08	0.42	−1.25
80	Licorice glycoside C2	Flavonoids	202657-55-4	1.38	0.21	0.49	−1.04
81	Lupa-1,20(29)-dien-3-one (Glochidone)	Terpenoids	N.F.	1.83	0.07	0.42	−1.25
82	Luteolin-7-O-rutinoside	Flavonoids	3563-98-2	1.34	0.17	0.50	−1.00
83	LysoPC 17:2	Lipids	N.F.	1.15	0.41	0.15	−2.77
84	LysoPC 18:1	Lipids	N.F.	1.03	0.42	0.39	−1.36
85	LysoPC 18:1(2n isomer)	Lipids	N.F.	1.11	0.41	0.38	−1.40
86	LysoPC 18:2	Lipids	N.F.	1.26	0.36	0.49	−1.03
87	LysoPC 18:3	Lipids	N.F.	1.19	0.39	0.44	−1.20
88	LysoPC 18:3(2n isomer)	Lipids	N.F.	1.13	0.40	0.45	−1.14
89	LysoPC 20:5	Lipids	162440-04-2	1.33	0.42	486.32	8.93
90	LysoPE 18:2(2n isomer)	Lipids	N.F.	1.06	0.41	0.45	−1.14
91	LysoPE 18:3	Lipids	N.F.	1.31	0.35	0.46	−1.14
92	LysoPE 18:3(2n isomer)	Lipids	N.F.	1.57	0.29	0.39	−1.35
93	Naringenin 7-Sulfate	Flavonoids	N.F.	1.21	0.27	0.34	−1.57
94	O-Phosphocholine	Alkaloids	107-73-3	1.73	0.11	0.42	−1.24
95	Pedalitin	Flavonoids	22384-63-0	1.09	0.40	2.32	1.21
96	Phe-Glu-Leu	Amino acids and derivatives	N.F.	1.96	0.03	0.35	−1.53
97	Pinocembrin-7-O-rutinoside	Flavonoids	118985-31-2	2.31	0.01	10.10	3.34
98	Protocatechuic Acid Methyl Ester	Phenolic acids	2150-43-8	2.10	0.02	2.45	1.29
99	Protocatechuic acid 1-O-Rutinoside	Phenolic acids	N.F.	1.85	0.21	0.41	−1.30
100	Protocatechuic acid 4-O-(2″-O-Galloy)Glucoside	Phenolic acids	N.F.	1.54	0.36	0.12	−3.08
101	Protocatechuic acid 4-O-(6″-O-Galloy)Glucoside	Phenolic acids	N.F.	1.43	0.38	0.13	−2.93
102	Prunetin-5-O-glucoside	Flavonoids	N.F.	1.47	0.16	2.01	1.01
103	Quercetin 5-O-p-coumaroyl rhamnoside	Flavonoids	N.F.	1.56	0.27	0.46	−1.12
104	Quercetin 7,3′,4′-trimethyl ether-3-glucoside	Flavonoids	N.F.	1.52	0.21	2.30	1.20
105	Quercetin 7-O-p-coumaroyl rhamnoside	Flavonoids	N.F.	1.15	0.39	0.48	−1.05
106	Quercetin-7-O-glucoside	Flavonoids	491-50-9	1.29	0.37	2.32	1.22
107	Shomaside F	Phenolic acids	N.F.	1.58	0.29	0.20	−2.35
108	Styrylamine	Alkaloids	83148-11-2	1.02	0.45	0.50	−1.00
109	Sulfatricalysine C	Others	N.F.	1.58	0.34	0.25	−2.02
110	Theaflagallin	Tannins	102208-15-1	1.94	0.12	2.37	1.25
111	Vnilloyltartaric acid	Phenolic acids	N.F.	2.26	0.02	2.16	1.11
112	Zizyberanalic acid	Terpenoids	N.F.	2.09	0.27	0.07	−3.94
113	[(8S,9R,10S,11R)-3,8,10-trihydroxy-4,5,19-trimethoxy-9,10-dimethyl-15,17-dioxatetracyclo [10.7.0.02,7.014,18]nonadeca-1(19),2,4,6,12,14(18)-hexaen-11-yl]acetate	Lignans and Coumarins	N.F.	1.24	0.35	2.24	1.17
114	alaschanioside C	Lignans and Coumarins	157543-21-0	2.12	0.05	2.08	1.06
115	clematichinenol	Lignans and Coumarins	N.F.	1.96	0.06	0.48	−1.05
116	orthosphenic acid	Terpenoids	86632-20-4	1.44	0.34	0.31	−1.69

Notes: CAS: Chemical Abstracts Service registry number; VIP: Variable Importance in Projection, which reflects the contribution of a metabolite to the OPLS-DA model; *p*-value: Probability value used to determine the statistical significance of the differences between groups; FC: Fold Change, representing the ratio of the average metabolite peak area between the high-altitude (HFT) and low-altitude (LFT) samples. N.F.: Not Found.

**Table 2:** The variation in different types of differential metabolites.

NO	Compounds	Type
	**Alkaloids**	
1	(14-Ethyl-4,6,19-trimethoxy-9,11-dioxa-14-azaheptacyclo [10.7.2.12,5.01,13.03,8.08,12.016,20]docosan-21-yl)acetate	down
2	(1s)-1-[(4-hydroxyphenyl)methyl]-7-methoxy-1,2,3,4-tetrahydroisoquinolin-8-ol	down
3	(E)-S-(3-(4-Hydroxy-3,5-dimethoxyphenyl)allyl)cysteine	down
4	O-Phosphocholine	down
5	Styrylamine	down
	**Amino acids and derivatives**	
6	Homoproline	up
7	Cis-L-3-hydroxyproline	down
8	D-Allo-Isoleucine	down
9	L-Homonorleucine	down
10	Phe-Glu-Leu	down
	**Flavonoids**	
11	2,3,5-trimethoxy-1-{[(2s,3r,4s,5s,6r)-3,4,5-trihydroxy-6-(hydroxymethyl)oxan-2-yl]oxy}xanthen-9-one	up
12	3′-Methoxydaidzin	up
13	3′-O-Methyl-6-hydroxygallocatechin 3-O-(2-Amino-5-hydroxypentanoate)	up
14	3-Hydroxyphloretin-4′-O-[4″,6″-O-(S)-HHDP]-β-D-glucoside	up
15	6-Hydroxyluteolin	up
16	Acacetin-7-O-galactoside	up
17	Carthamidin	up
18	Catechin glucosyl glucoside	up
19	Eupatilin-7-O-glucoside	up
20	Formononetin-7-O-glucoside (Ononin)	up
21	Pedalitin	up
22	Pinocembrin-7-O-rutinoside	up
23	Prunetin-5-O-glucoside	up
24	Quercetin 7,3′,4′-trimethyl ether-3-glucoside	up
25	Quercetin-7-O-glucoside	up
26	3,6-dihydroxy-4,5-dimethoxy-1-{[(3r,4s,5s,6r)-3,4,5-trihydroxy-6-(hydroxymethyl)oxan-2-yl]oxy}xanthen-9-one	down
27	Kaempferol 3-O-beta-D-(p-coumaroyl)-sambubioside	down
28	Kaempferol-3-O-(2″-galloyl)galactoside	down
29	Kaempferol-3-O-(6″-galloyl)galactoside	down
30	Kaempferol-3-O-(6″-galloyl)glucoside	down
31	Kaempferol-3-O-glucuronide-7-O-glucoside	down
32	Licorice glycoside C2	down
33	Luteolin-7-O-rutinoside	down
34	Naringenin 7-Sulfate	down
35	Quercetin 5-O-p-coumaroyl rhamnoside	down
36	Quercetin 7-O-p-coumaroyl rhamnoside	down
	**Lignans and Coumarins**	
37	Dalbergin glucoside	up
38	Isohydroxymatairesinol	up
39	[(8S,9R,10S,11R)-3,8,10-trihydroxy-4,5,19-trimethoxy-9,10-dimethyl-15,17-dioxatetracyclo [10.7.0.02,7.014,18]nonadeca-1(19),2,4,6,12,14(18)-hexaen-11-yl]acetate	up
40	alaschanioside C	up
41	Hydroxymethyl coumarin glucoside	down
42	Icariside E5	down
43	clematichinenol	down
	**Lipids**	
44	LysoPC 20:5	up
45	1-(((2-(dimethylamino)ethoxy)(hydroxy)phosphoryl)oxy)-3-hydroxypropan-2-yl (9Z,12Z,15E)-octadeca-9,12,15-trienoate	down
46	1-(2,3-dihydroxypropoxy)-3-(((2-(dimethylamino)ethoxy)(hydroxy)phosphoryl)oxy)propan-2-yl (8E,11Z,14Z)-octadeca-8,11,14-trienoate	down
47	1-Linoleoyl-2-Lysophosphatidic Acid Monobutylamine Ester	down
48	2-(2,3-dihydroxypropoxy)-3-(((2-(dimethylamino)ethoxy)(hydroxy)phosphoryl)oxy)propyl (8E,11Z,14Z)-octadeca-8,11,14-trienoate	down
49	2R-Hydroxyoctadecanoic Acid	down
50	3-(((2-(dimethylamino)ethoxy)(hydroxy)phosphoryl)oxy)-2-hydroxypropyl (9Z,12Z,15E)-octadeca-9,12,15-trienoate	down
51	3-(((2-(dimethylamino)ethoxy)(hydroxy)phosphoryl)oxy)-2-hydroxypropyl oleate	down
52	LysoPC 17:2	down
53	LysoPC 18:1	down
54	LysoPC 18:1(2n isomer)	down
55	LysoPC 18:2	down
56	LysoPC 18:3	down
57	LysoPC 18:3(2n isomer)	down
58	LysoPE 18:2(2n isomer)	down
59	LysoPE 18:3	down
60	LysoPE 18:3(2n isomer)	down
	**Nucleotides and derivatives**	
61	Flavin Single Nucleotide(FMN)	up
62	Guanosine 3′,5′-cyclic monophosphate	up
63	Guanine	down
64	Isoguanine	down
	**Organic acids**	
65	2-trans-abscisic acid D-glucosyl ester	up
66	3-Hydroxybutyric acid	up
67	Jasmonic acid	up
	**Phenolic acids**	
68	1-O-Caffeoyl-6-O-galloyl-β-D-glucose	up
69	1-O-Galloyl-3-O-Feruloyl-β-D-glucose	up
70	1-O-Galloyl-4-O-p-Coumaroyl-β-D-glucose	up
71	1-O-Galloyl-6-O-p-Coumaroyl-β-D-glucose	up
72	3-Hydroxycinnamic Acid	up
73	Protocatechuic Acid Methyl Ester	up
74	Vnilloyltartaric acid	up
75	1-Galloyl-6-O-Benzoyl Glucose	down
76	1-O-Caffeoyl-(6-O-glucosyl)-β-D-glucose	down
77	1-O-Caffeoylglycerol	down
78	1-O-p-Coumaroylsedoheptulose	down
79	3,4-di-hydroxyphenethyol alcohol 4-O-β-D-(6′-O-galloyol)-glucopyranoside	down
80	3-Hydroxybenzoic acid	down
81	4-Hydroxybenzoyl-1-O-(6″-O-galloyl)glucoside	down
82	4-O-Galloylarbutin	down
83	Dihydroxybenzoyl xyloside	down
84	Gallic acid-1-O-xyloside	down
85	Gallic acid-3-O-(6′-O-galloyl)glucoside	down
86	Isotachioside	down
87	Protocatechuic acid 1-O-Rutinoside	down
88	Protocatechuic acid 4-O-(2″-O-Galloy)Glucoside	down
89	Protocatechuic acid 4-O-(6″-O-Galloy)Glucoside	down
90	Shomaside F	down
	**Quinones**	
91	3,8-Dihydroxy-1-methoxy-9,10-anthraquinone glucoside	down
92	5,6-Dihydroxylucidin-3β-primeveroside	down
	**Tannins**	
93	Ellagic acid	up
94	Theaflagallin	up
95	1-Methoxybenzoyl-6-O-Galloyl-D-Glucose	down
	**Terpenoids**	
96	1-Trans-5-trans-germacrone	up
97	Ent-16α,17-Dihydroxykauran-2-one	up
98	Germacrone	up
99	1-Hydroxylup-20(29)-en-3-one (Glochidonol)	down
100	8-Epiloganic acid	down
101	Furanodienone	down
102	KakkaSaponin II	down
103	Lupa-1,20(29)-dien-3-one (Glochidone)	down
104	Zizyberanalic acid	down
105	orthosphenic acid	down
	**Others**	
106	(3R,4S,6R)-p-Menth-1-ene-3,6-diol-3-O-β-D-glucoside	up
107	D-Panthenol	up
108	Epigoitrin	up
109	Hydroxytyrosol	up
110	2,5,8-trimethylnona-1,4,7-triene-1,9-diol 1-O-Glyceryllinolenate	down
111	2-Methyl-3-hydroxyindan-1-one	down
112	3-Hydroxy-1-(4-Hydroxy-3-Methoxyphenyl)Propan-1-One	down
113	3-Hydroxy-5-Methoxyphenyl-6-O-Galloyl-Beta-D-Galactopyranoside	down
114	3-Hydroxy-5-Methylphenol-1-O-Glucoside	down
115	4-Hydroxyacetophenone	down
116	Sulfatricalysine C	down

**Table 3:** KEGG annotation and enrichment analysis.

KEGG_Pathway	ko_ID ^a^	Sig_Compound ^b^	Compound ^c^	Sig_Compound_All ^d^	Compound_All ^e^
Butanoate metabolism	ko00650	1	8	23	583
Metabolic pathways	ko01100	8	254	23	583
Phenylalanine metabolism	ko00360	1	14	23	583
Luteolin aglycones biosynthesis	MetMap105	2	31	23	583
Biosynthesis of flavones aglycones III	MetMap121	2	16	23	583
Arginine and proline metabolism	ko00330	1	14	23	583
Pantothenate and CoA biosynthesis	ko00770	1	8	23	583
Oxidative phosphorylation	ko00190	1	6	23	583
Riboflavin metabolism	ko00740	1	7	23	583
Biosynthesis of secondary metabolites	ko01110	2	152	23	583
Biosynthesis of cofactors	ko01240	1	51	23	583
Isoflavonoid biosynthesis	ko00943	1	8	23	583
Purine metabolism	ko00230	2	15	23	583
Nucleotide metabolism	ko01232	1	21	23	583
alpha-Linolenic acid metabolism	ko00592	1	8	23	583
Plant hormone signal transduction	ko04075	1	5	23	583
Biosynthesis of kaempferol aglycones I	MetMap113	3	34	23	583
Biosynthesis of kaempferol aglycones II	MetMap114	1	29	23	583
Glycerophospholipid metabolism	ko00564	1	3	23	583
Biosynthesis of flavones aglycones I	MetMap110	2	14	23	583
Biosynthesis of isoflavones aglycones II	MetMap112	1	10	23	583
Biosynthesis of quercetin aglycones II	MetMap116	1	19	23	583

^a^ ko_ID: The number of the path in the KEGG or MetMap database. ^b^ Sig_compound: The number of significantly different metabolites annotated by KEGG or MetMap in this pathway. ^c^ compound: The number of metabolites detected that belong to this pathway. ^d^ Sig_compound_all: The number of significantly different metabolites annotated by KEGG or MetMap. ^e^ compound_all: The number of metabolites annotated by KEGG or MetMap of all measured metabolites.

## Data Availability

Data will be made available on request.
